# Bacterial isolates from diabetic foot ulcers and their antimicrobial resistance profile from selected hospitals in Addis Ababa, Ethiopia

**DOI:** 10.3389/fendo.2022.987487

**Published:** 2022-08-31

**Authors:** Asegdew Atlaw, Habtamu Biazin Kebede, Abdurezak Ahmed Abdela, Yimtubezinash Woldeamanuel

**Affiliations:** ^1^ Department of Microbiology, Immunology and Parasitology, College of Health Sciences, Addis Ababa University, Addis Ababa, Ethiopia; ^2^ Department of Medical Laboratory Science, Debre Birhan College of Health Sciences, Debre Birhan, Ethiopia; ^3^ Department of Internal Medicine, College of Health Sciences, Addis Ababa University, Addis Ababa, Ethiopia

**Keywords:** diabetic Mellitus, diabetic foot ulcer, antimicrobial susceptibility testing, multidrug resistance, addis Ababa, ethiopia

## Abstract

**Introduction:**

Infected diabetic foot ulcer (IDFU) is a worldwide problem associated with diabetes mellitus. It could lead from soft tissue infection to bone infection and is a leading cause of lower limb amputation. Gram-negative and Gram-positive bacteria, including anaerobic bacteria and fungi, are considered potential causes of infection. The early diagnosis of DFU infection and appropriate treatment based on the identification of the pathogens and their antimicrobial susceptibility pattern is important for good prognosis. Therefore, the purpose of this study was to isolate the bacteria that infect foot ulcers in selected Hospitals and determine their antimicrobial resistance profile.

**Method:**

An institutional-based multicenter, cross-sectional study was conducted in selected Hospitals in Addis Ababa, Ethiopia, from November 2020 to May 2021. A sterile swab was used to collect samples from the foot ulcer and a sterile needle to collect pus. Isolates were identified by culture, Gram-staining, and a series of biochemical tests. For each bacterial species identified, the antibiotic profiling was determined by the Kirby-Bauer disk diffusion method.

**Results:**

one hundred and twenty-seven pathogenic bacteria were isolated from samples taken from 130 patients with a diabetic foot ulcer. Sixty-eight percent had growth of multiple microorganisms. Two-thirds (66.7%) of the isolates were gram-negative bacteria. The predominant bacterial species were *S. aureus* 25.19% (32/127), *Pseudomonas species* 18.89% (24/127), and *Escherichia coli* 16.53% (21/127). Overall, 92.9% (118/127) of the isolates were identified as multi-drug resistant. Gram-positive isolates were susceptible to chloramphenicol, clindamycin, and amikacin. Gram-negative isolates were also sensitive to chloramphenicol, aztreonam, and amikacin.

**Conclusion:**

The majority of bacteria isolated from patients presenting with Diabetic foot ulcer infections were found to be multi-drug resistant in the study sites of the current study. The results demonstrate the importance of timely identification of infection of diabetic foot ulcers, proper sample collection for identification of the pathogens and for determining their antibiotic susceptibility pattern before initiating antimicrobial treatment

## Introduction

Diabetic foot ulcer (DFU) is a common complication in diabetes and can lead to a considerable social, psychological, and economic burden on patients and the health sector (1). DFU accounts for significant morbidity and mortality. DFU patients have a 2.5 times higher risk of death compared to diabetic patients without foot ulcers (2, 3). According to the International Diabetes Federation, 9.1-26.1 billion people will develop DUFs every year (1). The prevalence of DFU has increased significantly with a recent global prevalence averaging around 6.4% (4).

Once DFU occurs, the main challenge is the increased vulnerability to different potential pathogens with potentially serious outcomes such as infection, gangrene, osteomyelitis, amputation, and even death (5). According to IDSA and other studies, infection with diabetic foot ulcers will increase the chance of amputation by 50% compared to patients with uninfected foot ulcers (6, 7).

Diabetes**-**related hospital admissions are most pronounced because of infected diabetic foot ulcers. Major and minor (83% and 96% respectively) amputations have been done related to DFU aggravated by infection (8). On the other hand, diabetic foot osteomyelitis development is seen in around 44**-**68% of patients admitted to the hospital and it is the principal reason for amputation among such patients (9).

A study from Nigeria revealed that the burden of diabetic foot ulcers accounts for 24.9% and that the majority of the ulcers are already infected with Wagner grade ≥ 3 (10).

Ethiopia is also among the countries affected by diabetic foot ulcers. A systematic meta-analysis indicated that the national prevalence of DFU in Ethiopian diabetic patients was 11.27% (11). Other related studies in Ethiopia, have shown the diverse prevalence in the country as 17.9% in Nekemte (12), 13.6% in Gondar (13), and 15% in Arbaminch (14), and 78% in Addis Ababa (15). In the study from Addis Ababa, DFU with cellulitis was 12.2%, DFU with toe gangrene at 16.3%, DFU with foot gangrene at 18.4%, and only 31.1% with foot ulcer (15). Another more recent study found that the rate of foot ulcers at Tikur Anbessa Specialized Hospital was 26% (16).

Diabetic foot ulcer infection is essentially polymicrobial. Many complications of DFUs are caused by bacterial infections and can be minimized by paying due attention to the identification and control of infections. It is necessary to detect the specific pathogens and their susceptibility pattern to initiate early treatment with the appropriate antibiotics.

Therefore, this study aimed to identify the bacteria that cause diabetic foot ulcers and the antimicrobial sensitivity patterns of these bacterial isolates.

## Materials and methods

A multicenter hospital**-**based cross**-**sectional study was done in the diabetes mellitus (DM) clinics of three selected public hospitals in Addis Ababa, Ethiopia. The study sites were Tikur Anbessa Specialized Hospital (TASH), Menelik II, and Yekatit 12 Hospitals. All adult diabetic patients with diabetic foot ulcers, whose ulcers were greater than or equal to the Wagner first degree grading system, who visited the DM clinics at the study sites during the study period, and gave informed consent were included in the study. The study took place from November 2020 to May 2021.

Samples were taken from the deepest part of the ulcer using two sterile swabs, soaked in sterile glucose broth. The samples were taken using a firm circular motion with the swab. One swab was used for Gram staining and the other was used for culture. Semi-structured questionnaires were used to collect sociodemographic and other clinical data.

A Gram smear directly from the sample was examined. The samples were inoculated on blood agar, MacConkey agar, and chocolate agar. The inoculated plates were incubated at 37°C overnight and the plates were examined for growth the next day.

In this study, ulcers were classified according to the Wagner Diabetic Foot Ulcer Classification System. The classification was Grade 0-Pre-ulcerative, with no open lesion or cellulitis, Grade1-Superficial ulcer, Grade2-Deep ulcer up to tendons and joint tissue, Grade 3-Deep ulcer with abscess, osteomyelitis, and joint sepsis, Grade 4- Localized gangrene of forefoot or heel, and Grade 5-Gangrene of entire foot/global gangrene.

### Antimicrobial susceptibility testing

Antimicrobial susceptibility testing (AST) was done for the isolated bacteria with 21 antibiotics on Mueller Hinton Agar (MHA) using the Kirby Bauer disk diffusion technique according to the Clinical Laboratory Standard Institute (CLSI) guidelines 2020 (17). The inoculum for each isolate was prepared by emulsifying colonies from the purified culture overnight in normally sterile saline (0.85%) in test tubes with turbidity adjusted to standard 0, 5 McFarland. The bacterial suspension was spread evenly on the MHA plate with a sterile swab, left for 3 minutes, and then the antibiotic discs were applied.

For Gram**-**negative bacteria the following antibiotic discs were used (in/disk): ampicillin**-**sulbactam (10/5), amoxicillin and clavulanic acid (10/10 μg), ceftriaxone (18), cefotaxime (18), cefoxitin (18), ceftazidime (18), cefepime (18), amikacin (18), gentamicin (19), ciprofloxacin (5), sulfamethoxazole**-**trimethoprim(1.25/23.75), piperacillin**-**tazobactam (100/10), tobramycin (18), imipenem (19), aztreonam (18), and meropenem (19). While for Gram**-**positive bacteria, antibiotics discs used were (in μg/disk): gentamicin (19), doxycycline (18), erythromycin (14), vancomycin (18), cefoxitin (18), sulfamethoxazole**-**trimethoprim (1.25/23.75), ciprofloxacin (5) penicillin (10 units), clindamycin (2), and chloramphenicol (18) (17).

The plates were incubated at 35°C for 16**-**18 hours, and the diameters of the zone of inhibition were measured with a Vernier caliper, and the results were interpreted according to the CLSI standards (17).

Standard operating procedures (SOPs) were used for specific purposes for all laboratory procedures. Quality control strains of *Escherichia coli* ATCC^®^ 25922, *Enterococcus faecalis* ATCC^®^ 29212, *Pseudomonas aeruginosa* ATCC^®^ 27853, *Staphylococcus aureus* ATCC^®^ 25923, *K. pneumoniae* ATCC^®^1705, and *K. pneumoniae* ATCC^®^1706 were used to confirm the result of antibiotics, media and to assess the quality of the general laboratory procedure (17). The quality of the reagents, antibiotic disk, and media used was checked regularly.

### Ethical consideration

Ethical approval was obtained from the ethics and review committee of the Department of Microbiology, Immunology, and Parasitology, College of Health Sciences, Addis Ababa University (meeting DERC/0022/2020). A formal letter of support from the Department was written to the three study sites. An information sheet with the necessary information about the study was given to the potential participants and their voluntary consent was taken before enrolling them in the study. For those who were younger than 18, their parents or guardians were asked for consent and assent taken from the children. Confidentiality was maintained by omitting their personal identifiers throughout the study.

## Results

### Sociodemographic and clinical characteristics of participants

One hundred thirty (130) study participants (23 from TASH, 31 from Yekatit-12 Hospital, and 76 from Menelik II hospital) were included in the present study. Out of these, 88 (67.69%) were men and 42 (32.3%) were women. Most participants were between 50 and 75 years old and the mean age of the study, participants were 54 ±  7SD. The majority of the study participants were residents of Addis Ababa (84.5%), while only 15.5% were from rural areas The majority of the participants had type I diabetes as tabulated in **Table 1**.

**Table 1:** Sociodemographic and clinical characteristics of the study participants.

Characteristics	Categories	Frequency (n)	(%)
Sex	Male	89	68.2
	Female	41	31.8
Age	<40	4	3.2
	41**-**50	36	27.2
	51**-**60	42	32.7
	61**-**70	30	29.5
	71-80	17	14.4
	≥ 81	1	0.8
	Total	130	100
Number of participants at study sites	TASH	23	17.69
	Yekatit**-**12 hospital	31	23.84
	Menelik**-**II hospital	76	58.46
Residence	Urban	110	84.61
	Rural	20	15.38
Type of diabetes	Type**-**I	67	51.53
	Type-II	63	48.46
Duration of diabetes	< 1 year	0	0
	1**-**10 years	75	53.38
	11**-**20 years	38	27.69
	21**-**30 years	16	12.30
	>31 years	1	0.76
HGBA1C	1.61**-**8.06 mmol/mol	0	0
	9.67-16.11 mmol/mol l	75	57.69
	17.72**-**24.17 mmol/mol l	43	33.07
	≥24.17 mmol/mol	2	1.53
Hypertension	Yes	70	53.84
	No	60	46.15
Kidney disease	Yes	24	18.46
	No	106	81.53
Peripheral Neuropathy (PN)	Yes	104	80
	No	26	20
PVD	Yes	43	33.07
	No	87	66.92
Leg skin texture	Dry skin	40	30.76
	Moist skin	53	40.7
	Cracked skin	37	28.46
Wagner**’**s classification system	Grade 1	3	2.3
	Grade 2	45	34.61
	Grade 3	62	47.69
	Grade 4	19	14.61
	Grade 5	1	0.76
** Total**		130	100

PVD, Peripheral Vascular Disease; HGBA1C, Hemoglobin A1C.

### The magnitude of bacterial isolates from diabetic foot ulcer infections

One hundred and twenty-seven bacterial isolates were identified from 130 patients with diabetic foot ulcers. Of these, 41 (32%) were Gram-positive, and 86 (68%) were Gram-negative isolates. Sixty-eight percent (82/120) of the samples had poly-bacterial growth. The percentage of Gram-negative bacteria 86 (68%) was greater than Gram-positive bacteria 41 (32%). Among the isolated bacteria, the most predominant bacteria were *Staphylococcus aureus* 25.19% (32/127), followed by *Pseudomonas species* 18.89% (24/127), and *Escherichia coli* 16.53% (21/127). Other isolates included *Acinetobacter species* (9.4%), *Klebsiella pneumoniae* (7.9%), *Serratia species* (4.7%), *Enterococcus species* (3.1%), *Proteus vulgaris* (3.1%), and *Proteus mirabilis* (3.1%) (**Table 2**).

**Table 2:** Bacterial isolates among diabetic foot ulcer study participants.

Bacterial profiles		Frequency	Percentage
**Number of isolates per sample/case**	Mono**-**bacterial	38	31.66
	Poly**-**bacteria	82	68.33
**Bacterial isolates**	*S. aureus*	32	25.19
	*Pseudomonas species*	24	18.89
	*Escherichia coli*	21	16.53
	*Acinetobacter species*	12	9.44
	*Klebsiella pneumoniae*	10	7.87
	Serratia	6	4.72
	*Enterococcus species*	4	3.14
	*Proteus vulgaris*	4	3.14
	*Proteus mirabilis*	4	3.14
	*Citrobacter species*	4	3.14
	*Viridans streptococcus spp*	3	2.36
	*Streptococcus pyogenes*	2	1.57
	*Klebsiella oxytoca*	1	0.78
	Total isolates	127	100

The highest number of culture-positive cases 50.3% (64/127), was found in Wagner grade 3 of diabetic foot ulcers, followed by Wagner grade 2 26.7% (34/127). With an increase in Wagner’s score, the rate of infections caused by gram-negative bacteria increased. *Staphylococcus aureus* 32 (25.1%), *Pseudomonas species* 24 (18.8%), and *E. coli* (16.5%) were the most common isolates in each of Wagner’s rating scales. Overall, infection was caused by one bacteria in 31.66% (38/120) and polymicrobial in 68.33% (82/120) samples. In general, as the degree of ulcers increases, the percentage of ulcers with polymicrobial growth increases (**Table 3**).

**Table 3:** Distribution of bacterial isolates among Wagner classification of DFUs system.

Isolated Bacteria	Wagner classification of DFUs, n (%)					Total
	Grade 1	Grade 2	Grade 3	Grade 4	Grade 5	
*Acinetobacter species*	0	0	10(83.3)	2 (16.6)	0	12 (9.4)
*Citrobacter species*	0	1 (25)	2 (50)	1 (25)	0	4 (3.1)
*E.coli*	0	4 (19)	9 (42.8)	7 (33.3)	1 (4.7)	21 (16.5)
*Enterococcus species*	0	2 (50)	2 (50)	0	0	4 (3.1)
*K. oxytoca*	0	0	0	1	0	1 (0.8)
*K. pneumoniae*	0	1 (10)	5 (50)	4 (40)	0	10 (7.8)
*Proteus mirabilis*	0	2 (50)	1 (25)	1 (25)	0	4 (3.1)
*Proteus vulgaris*	0	2 (50)	0	2 (50)	0	4 (3.1)
*Pseudomonas species*	0	7 (29.1)	13(54.1)	4 (16.6)	0	24 (18.8)
*S. aureus*	1 (3.1)	11(34.3)	18(56.2)	2 (6.25)	0	32 (25.1)
*S. pyogenes*	1 (50)	0	1 (50)	0	0	2 (1.5)
*Serratia*	0	3 (50)	2 (33.3)	1 (16.6)	0	6 (4.7)
*Viridian streptococcus species*	0	1(33.3)	1 (33.3)	1 (33.3)	0	3 (2.3)
**Total**	2(1.5)	34(26.7)	64(50.3)	26(20.4)	1(0.8)	127 (100)

### Antimicrobial susceptibility profiles of gram-positive isolates

As shown in **Table 4**, among 41/127 (32.2%) Gram-positive bacteria, all *S. aureus* and *Enterococcus species* were resistant to oxacillin, penicillin, cefoxitin, and bacitracin. However, a lower level of 18.7% (6/32) susceptibility rates for *S. aureus* isolates were documented for oxacillin. A high level of resistance was also observed among the majority of the isolates of S*. aureus* and all *Enterococcus species*, which were resistant to gentamycin, doxycycline, erythromycin, and cotrimoxazole. However, all *S. pyogenes* and Viridans streptococcus species were sensitive to the majority of antimicrobial agents.

**Table 4:** Antimicrobial susceptibility patterns of isolated gram-positive bacteria.

Antimicrobial tested		Susceptibility and resistance pattern of isolated Gram-positive bacteria N (%)						
Classes	Antibiotics	*S. aureus* (N = 32)		*Enterococcus spp* (N = 4)		*S. pyogenes* (N = 2)	*Viridian streptococcus spp *(N = 3)	
		R	S	R	S	S	R	S
Penicillin	PEN	32 (100)	0	4 (100)	0	2 (100)	1 (33.3)	2 (66.6)
	OX	26 (81.2)	6 (18.7)	4 (100)	0	2 (100)	1 (33.3)	2 (66.6)
Cephamycin	CXT	26 (81)	6 (19)	4 (100)	0	2 (100)	1 (33.3)	2 (66.6)
Aminoglycosides	GEN	25 (78)	7 (22)	4 (100)	0	2 (100)	2 (66.6)	1 (33.3)
Aminoglycosides	AMK	6(18)	26 (82)	2 (100)	2 (50)	–	0	3 (100%)
Tetracycline	DO	26 (81.3)	6 (18.7)	4 (100)	0	–	2 (66.6)	1 (33.3)
Quinolones	CPR	16 (50)	16 (50)	3 (75)	1 (25)	2 (100)	1 (33)	2 (67)
Bacitracin	BC	32 (100)	0	4 (100)	0	2 (100)	3 (100)	0
Sulfonamides	SXT	21 (87.5)	3 (12.5)	3 (75)	1 (25)	2 (100)	0	3 (100)
Chloramphenicol	CHL	0	32 (100)	0	4(100)	2 (100)	0	3 (100)
Macrolides Lincosamides	ER	22 (68.8)	10 (31.2)	4 (100)	0	2(100)	0	3(100)
	DA	12 38)	20 (62)	3 (75)	1 (25)	2 (100)	0	3 (100)
Glycopeptide	VA	12 (37)	20 (63)	2 (50)	2 (50)	2 (100)	0	3 (100)
Total		198 (56.6)	152 (43.4)	41 (78.8)	11 (21.2)	22 (100)	11 (28.2)	28 (71.8)

Keys: PEN, Penicillin; OX, Oxacillin; CXT, Cefoxitin; GEN, Gentamycin; AMK, Amikacin; DO, Doxycycline; CPR, Ciprofloxacin; BC, Bacitracin; SXT, TTrime; CHL, Chloramphenicol; ER, Erythromycin; DA, Clindamycin; VA, Vancomycin.

The majority of *S. aureus* isolates were sensitive to amikacin 81.25% (26/32), ciprofloxacin 50% (16/32), clindamycin 62.5% (20/32), and vancomycin 62.5% (20/32). All isolated *S. aureus* and *Enterococcus species* were sensitive to chloramphenicol (100%). In this study, 50% (2/4) of *Enterococcus species* were resistant to vancomycin. Overall, 56.4% of *S. aureus* isolates, *78.8*% of *Enterococcus species* isolates, and 72% of *Viridans streptococcus species* were resistant to the tested antibiotics (**Table 4**).

### Antimicrobial-resistance profiles of the gram-negative isolates

All the gram-negative isolates were resistant to cefoxitin, ampicillin-sulbactam, tobramycin, polymyxin b, cefepime, and augmentin. *Acinetobacte*r and *Pseudomonas* species were the most resistant to most antibacterial drugs, including amikacin, chloramphenicol, aztreonam, ceftriaxone, ceftazidime, imipenem, and meropenem. More than half of gram-negative bacterial isolates were resistant to doxycycline, trimethoprim, piperacillin, tazobactam, ceftriaxone, cefotaxime, imipenem, and meropenem.

The majority of *Escherichia coli*, *Klebsiella pneumoniae*, Serratia, *Proteus vulgaris*, *Proteus mirabilis*, *Citrobacter species*, and *Klebsiella oxytoca*, were sensitive to Chloramphenicol, Amikacin, and Ceftazidime. Amikacin was found to be the best drug for *Citrobacter species* (**Table 5**).

**Table 5:** Resistance pattern of gram-negative isolates.

Antibiotic tested		Resistance pattern of isolated Gram-negative bacteria n (%)							
Classes	Antibiotics	*Acinetobacter spp*(N = 12)	*Pseudomonas spp*(N = 24)	*K.P*(N = 10)	*E.coli*(N = 21)	Serratia(N = 6)	*P. mirabilis* (N = 4)	*P. vulgaris*(N = 4)	*Citrobacter spp*(N = 4)
Aminoglycosides	AMK	10 (83.3)	11 (45.8)	5 (50	5 (28.8)	2(33.3)	2 (50)	2 (50)	0
	TOB	12 (83.3)	21 (87.5)	7 (70)	19 (90.5	5 (83.3)	4 (100)	3 (75)	3 (75)
Tetracycline	DO	11 (91.7)	23 (95.8)	10 (100)	18 (86)	6 (100)	4 (100)	3 (75)	3 (75)
Quinolones	CPR	8 (66.7)	13 (54.2)	4 (40)	8 (38.0)	5(83.3)	2 (50)	2 (50)	2 (50)
Sulfonamides	SXT	10 (83.3)	21 (87.5)	9 (90)	19 (90)	5(83.3)	2 (50)	4 (100)	4 (100)
Chloramphenicol	CHL	8 (66.7)	16 (66.7)	3 (30)	2 (9.5)	6(100)	2 (50)	0	2 (100)
Monobactam	ATM	7 (58.3)	13 (54.2)	2 (20)	5 (28.8)	2 (33.3)	2 (50)	3 (75)	0
Polymyxin	PB	12 (100)	24 (100)	10 (100)	21 (100)	6 (100)	4 (100)	4 (100)	4 (100)
Cephalosporins	CXT	11 (91.6)	24 (100)	10 (100)	20 95.2)	6(100)	4(100)	4(50)	4(100)
	CFP	12 (100)	24 (100)	10 (100)	21 (100)	6 (100)	4 (100)	4 (100)	4 (100)
	CTR	12 (100)	20 (83.3)	6 (60)	11 (52)	6 (100)	3 (75)	1 (25)	2 (50)
	CTX	11 (8.3)	21 (87.5)	8 (80)	15 (74)	6 (100)	3 (75)	1 (25)	3 (75)
	CAZ	7 (58.3)	13 (54.2)	4 (40)	7 (33.3)	3 (33)	3 (75)	0	0
Beta-lactam Inhibitors	TZP	6 (50)	16 (66.7)	4 (40)	7 (33.3)	2(33.3)	2 (50)	0	2 (50)
	SAM	12 (100)	24 (100)	10 (100)	21 (100)	6 (100)	4 (100)	4 (100)	4 (100)
	AUG	12 (100)	24 (100)	10 (100)	21 (100)	6 (100)	4 (100)	4 (100)	4 (100)
Carbapenem	IMI	6 (50)	11 (45.8)	6 (60)	5 (23.8)	4 (66.6)	1 (25)	0	0
	MER	7 (58.3)	9 (37.5)	4(40)	6 (28.5)	3 (50)	1(25)	1(25)	0

AMK, Amikacin; TOB, Tobramycin; DO, Doxycycline; CPR, Ciprofloxacin; SXT, Trimethoprim-sulfamethoxazole; FEP, Cefepime; TZP, Piperacillin-Tazobactam; SAM, Ampicillin-Sulbactam; AUG, Augmentin; CTR, Ceftriaxone; CTX, Cefotaxime; CAZ, Ceftazidime; IMI, Imipenem; MER, Meropenem; ATM, Aztreonam.

### The magnitude of multidrug-resistant isolates

Multidrug-resistance profiles of the organisms showed that of the 127 bacterial isolates, 92.9% (118/127) were MDR, that is resistant to more than two agents of antibiotic classes, whereas 7.08%(9/127) were non-MDR. Twenty-eight (88%) of *Staphylococcus aureus* isolates were MDR. *Acinetobacter* and *Pseudomonas* pathogens were resistant to all types of antibiotics. However, the MDR profiles within species vary (**Table 6**).

**Table 6:** Antibiogram of bacterial isolates.

Bacterial isolates	Antibiogram pattern, N (%)											
	R_0_	R_1_	R_2_	R_3_	R_4_	R_5_	R_6_	R_7_	R_8_	R_9_	R_10_	MDR
*S. aureus*	0	1 (3.1)	3 (9.3)	4 (12.5)	8 (25)	5 (15.6)	5 (15.6)	3 (9.3)	3 (9.3)	0	0	28 (87.5)
*Enterococcus species*	0	0	0	0	0	0	0	1 (25)	0	3 (75)	0	4 (100)
*S. pyogenes*	2 (100)	0	0	0	0	0	0	0	0	0	0	0
*Viridian species*	0	0	0	1 (33.3)	1 (33.3)	1 (33.3)	0	0	0	0	0	3 (100)
*Pseudomonas species*	0	0	2 (8.3)	3 (12.5)	3 (12.5)	4 (16.6)	5 (20.8)	0	6 (25)	1 (4.1)	0	22 (91.6)
*E.coli*	0	0	0	6 28.5)	6 (28.5)	4 (19.0)	3 (14.2)	1(4.7)	1 (4.7)	0	0	21 (100)
*Acinetobacter species*	0	0	0	0	2 (16.6)	2 (16.6)	2 (16.6)	1(8.3)	2 (16.6)	3 (25)	0	12 (100)
*K. pneumoniae*	0	0	0	0	2 (20)	4 (40)	2 (20)	0	1 (10)	1 (10)	0	10 (100)
*Serratia*	0	0	0	0	0	0	4(66.6)	2 (33.3)	0	0	0	6 (100)
*Proteus mirabilis*	0	0	0	1(25)	0	0	2 (50)	1 (25)	0	0	0	4 (1000
*Proteus vulgaris*	0	0	0	0	2 (50)	1 (25)	1 (25)	0	0	0	0	4 (100)
*Citrobacter species*	0	0	1 (25)	1 (25)	0	1 (25)	1 (25)	0	0	0	0	3 (75)
*K.oxytoca*	0	0	0	0	1	0	0	0	0	0	0	1
*Total*	2 (1.6)	1 (0.7)	6 (4.72	16 (12.5	25 (19.6)	22 (17.3)	25 (19.6)	9 (7.08)	13 (10.2)	8 (6.2)	0	118 (92.9)

Sensitive for all classes of antibiotics (R_0_), Resistance for one class of antibiotics (R_1_), Resistance for two classes of antibiotics (R_2_), Resistance for three classes of antibiotics (R_3_), Resistance for four classes of antibiotics (R_4_) etc., MDR-Multidrug-resistance.

## Discussion

Diabetic foot ulcers are the main complication of Diabetes mellitus. Diabetic foot ulcers, if left untreated, can become infected and cause other complications, such as gangrene, osteomyelitis, and amputation. Surgery and antibiotic therapy are the options used to control this infection. This study was conducted to determine the main pathogenic bacterial infections associated with DFU and their antimicrobial susceptibility patterns to commonly used antibiotics at the study sites.

A male predominance in the study participants was noted in this study, in line with previous studies in Indonesia and India (20, 21). This may be explained by the more active role of men in outdoor activities leading to injuries and exposure to the development of ulcers. Similarly, the majority of participants with DFU infections were found to be within the age range of 51**-**60 years in agreement with the same studies in India and Indonesia (20, 22).

In this study, ulcers were classified according to the Wagner Diabetic Foot Ulcer Classification System. The most common was grade 3 at 47.69% (62/130) followed by grade 2 at 34.61% (45/130), which is consistent with the research conducted in Egypt, where grade 3 was found in 50% (60/120) followed by grade 2 in 25% (30/120) of participants (23). Contrary to these findings, a study from India showed that grade 2 (69.2%) is higher than grade 3 (5.1%).

In this study, a high growth rate (92.3%) of the bacteria was found, comparable with an earlier study in Ethiopia with a growth rate of 77.3% (92/119), compared to no growth at 22.7% (27/1190) (15). A recent study also reported, that the growth rate was 81.7% (98/120), and no growth of 22% (18.34%), respectively (23).

Overall, gram-negative bacteria (71.6%) were predominantly isolated (86/120) compared to gram-positive isolates (34.16%). This finding is in agreement with an earlier study done in the same study site (out of three included in the current study) Tikur Anbessa Specialized Hospital, where gram-negative bacteria were isolated in 88.55% (54/61) versus 7% (11.47%) gram-positive bacteria (15). Similarly, a study from Egypt reported 56% gram-negative and 27.7% gram positives, while 79% gram-positive and 21% gram negatives were isolated in northeast India (23, 24).

The rate of bacterial isolation and the type of bacteria in the ulcer increased as the severity of the ulcer increased. This shows the extent to which organisms influence the DFU healing process, which is supported by various papers published elsewhere, such as in Nigeria (18), China (25), and India (21, 26).

In the present study, *S. aureus* was the predominant isolate 25.19% (32/127), unlike a previous study in Ethiopia which reported, *Klebsiella species* 23.9% (22/92) as the predominant bacteria followed by *Proteus species* 18.47% (17/92) (15). In Egypt*, P. mirabilis* (16.8%) is the most common isolate (24), in Saudi Arabia Pseudomonas *species* 15.6% (n = 134) (27), and in South America Pseudomonas *species* (18.8%) was the most common isolate (28). Similarly in agreement with studies in Kenya 17.5% (14/80 (29), Nigeria 32.9% (32/97 (30), India 24.42% (32/131) (31), 25% (18/85) (21), China 65.2% (n=232) (25), and in Iran 28% (n=92) (32). This shows that the predominant bacteria causing DFU infections could vary in different settings.

The current study found that the most common gram-negative isolates were *Pseudomonas species* (18.89%), followed by *Escherichia coli*, comparable with other studies conducted in Libya 17.5% (21/120) (33), India 23.2% (n = 85) (26) and 23.6% (n = 85) (21).

On the other hand, a previous study in Ethiopia reported that no *Pseudomonas species* were isolated from 92 cultured samples, whereas *E.coli* was isolated in 5.43% (5/92) (15). Similarly in Pakistan, the most common gram**-**negative bacteria was *E.coli* 15.72% (n=671) (34). This variation may be due to the sample size differences of the different studies and other unique characteristics of each study site.

A very high rate of multidrug resistance (92.9%) was found in the present study, consistent with the findings of a study in India (26), and Nigeria (35).

In this study, the majority of isolated *Staphylococcus aureus* were resistant to gentamicin; doxycycline, erythromycin, and trimethoprim, while the majority are sensitive to amikacin, oxacillin, ciprofloxacin, clindamycin, and vancomycin. Similarly, all enterococci are resistant to gentamicin, doxycycline, erythromycin, and trimethoprim. While all *Staphylococcus aureus* and Enterococci were found to be susceptible to chloramphenicol (100%) (36). The possible reasons for this high level of resistance could be multifactorial. It could be explained by inappropriate antibiotic use, self -medication, repeated courses of antibiotics associated with the chronic nature of the DFU, and the patients encounters with the Hospital environment during their frequent follow up visits.

The current study confirmed that both gram**-**positive and gram**-**negative aerobic pathogenic bacteria cause DFU infection in the study sites and because of their antimicrobial resistance profile can cause challenges for the management of patients and can lead to more complications such as osteomyelitis, and possibly amputation of the limbs.

### Limitation of the study

The current study, which has been done in a resource constrained environment has tried to address the problem of resistance to commonly used antibiotics in the study sites, but has its limitations. The study was done during the COVID-19 pandemic, which has affected the health seeking behavior of potential study participants. The study focused on anaerobic bacteria, even though naerobic bacteria are known to be important etiologic agents in DFU infections, including fungi. We were not able to do further analysis of the study using molecular techniques to get more information about the profile of the organisms isolated from these patients, because of resource constraints.

## Conclusion

Diabetic foot ulcers can be infected with a wide variety of pathogens and a large number of multi-drug resistant bacteria. In this study, *Staphylococcus aureus* was the dominant isolate followed by other gram-negative bacteria. In the current study, a high level of resistance to commonly used antibiotics was found highlighting the need for cautious care in the use of antibiotics for the treatment of infections. Some isolates in the current study were more sensitive to chloramphenicol, aztreonam, amikacin, clindamycin, and vancomycin, which can be used as first-line treatment for these infections. The results showed an overall increase in the resistance of bacteria to antimicrobial agents and emphasizes the importance of microbiological analysis and antimicrobial susceptibility testing before initiating antibiotics treatment for diabetic foot ulcer infections.

## Data availability statement

The original contributions presented in the study are included in the article/supplementary material. Further inquiries can be directed to the corresponding author.

## Ethics statement

The studies involving human participants were reviewed and approved by the Review and Ethics Committee of the Department of Microbiology, Immunology & Parasitology, College of Health Sciences, Addis Ababa University. The patients/participants provided their written informed consent to participate in this study.

## Author contributions

AA collected the primary data, conducted the analyses, and drafted the manuscript. HK and YW contributed to conceptualization, collection of data and data analysis. AA, YW, HK, and AAb have read, reviewed, and approved the article.

## Funding

The study was supported by funds from Addis Ababa University, Ethiopia.

## Acknowledgments

The authors would like to acknowledge the data collectors, particularly health extension workers. In addition, we are grateful to the study participants for their voluntary participation in the study. Moreover, the authors would like to acknowledge the following individuals, Shemse Sebre, Aminu Seman, and Shambel Araya for their assistance.

## Conflict of interest

The authors declare that the research was conducted in the absence of any commercial or financial relationships that could be construed as a potential conflict of interest.

## Publisher’s note

All claims expressed in this article are solely those of the authors and do not necessarily represent those of their affiliated organizations, or those of the publisher, the editors and the reviewers. Any product that may be evaluated in this article, or claim that may be made by its manufacturer, is not guaranteed or endorsed by the publisher.

## Abbreviations

DFU, Diabetic foot ulcer; DM, Diabetic Mellitus; ESBL,Extended spectrum; MDR,Multidrug resistance.

## References

[1] Armstrong D Boulton M Bus S. Diabetic foot ulcers and their recurrence. N Engl J Med (2017) 376(24):2367–75. doi: 10.1056/NEJMra1615439 28614678

[2] Chammas NK Hill RL Edmonds ME. Increased mortality in diabetic foot ulcer patients: The significance of ulcer type. J Diabetes Res (2016) 7. doi: 10.1155/2016/2879809 PMC4860228 27213157

[3] Walsh JW Hoffstad OJ Sullivan MO Margolis DJ. Association of diabetic foot ulcer and death in a population-based cohort from the united kingdom. Diabetes Med (2016) 33(11):1493–8. doi: 10.1111/dme.13054 26666583

[4] Zhang P Lu J Jing Y Tang S Zhu D Bi Y. Global epidemiology of diabetic foot ulceration: a systematic review and meta-analysis †. Ann Med (2017) 49(2):106–16. doi: 10.1080/07853890.2016.1231932 27585063

[5] Hitam SAS Hassan SA Maning N. The significant association between polymicrobial diabetic foot infection and its severity and outcomes. Malays J Med Sci (2019) 26(1):107–14. doi: 10.21315/mjms2019.26.1.10 PMC6419864 30914898

[6] Lipsky BA Berendt AR Cornia PB Pile JC Peters EJ Armstrong DG. Infectious diseases society of America clinical practice guideline for the diagnosis and treatment of diabetic foot infections. Clin Infect Dis (2012) 54(12):e132–73. doi: 10.1093/cid/cis346 22619242

[7] Noor S Zubair M Ahmad J. Diabetic foot ulcer–a review on pathophysiology, classification and microbial aetiology. Diabetes Metab Syndr (2015) 9(3):192–9. doi: 10.1016/j.dsx.2015.04.007 25982677

[8] Hicks CW Selvarajah S Mathioudakis N Sherman RE Hines KF Black JH 3rd. The burden of infected diabetic foot ulcers on hospital admissions and costs. Ann Vasc Surg (2016) 33:149–58. doi: 10.1016/j.avsg.2015.11.025 PMC6048950 26907372

[9] van Asten SA La Fontaine J Peters EJ Bhavan K Kim PJ Lavery LA. The microbiome of diabetic foot osteomyelitis. Eur J Clin Microbiol Infect Dis (2016) 35(2):293–8. doi: 10.1007/s10096-015-2544-1 PMC4724363 26670675

[10] Ugwu E Adeleye O Gezawa I Okpe I Enamino M Ezeani I. Burden of diabetic foot ulcer in Nigeria: Current evidence from the multicenter evaluation of diabetic foot ulcer in Nigeria. World J Diabetes (2019) 10(3):200–11. doi: 10.4239/wjd.v10.i3.200 PMC6422858 30891155

[11] Mulugeta H Wagnew F Zeleke H Tesfaye B Amha H Leshargie CT. Prevalence of diabetic foot ulcer and its association with duration of illness and residence in Ethiopia: a systematic review and meta-analysis. medRxiv (2019), 19003061. doi: 10.1101/19003061

[12] Bekele F Fekadu G Bekele K Dugassa D. Incidence of diabetic foot ulcer among diabetes mellitus patients admitted to nekemte referral hospital, Western Ethiopia: a prospective observational study. Endocrinol Metab Syndr (2019) 8(2):1–5.

[13] Mariam TG Alemayehu A Tesfaye E Mequannt W Temesgen K Yetwale F. Prevalence of diabetic foot ulcer and associated factors among adult diabetic patients who attend the diabetic follow-up clinic at the university of gondar referral hospital, north West Ethiopia, 2016: Institutional-based cross-sectional study. J Diabetes Res (2017), 2879249. doi: 10.1155/2017/2879249 28791310 PMC5534295

[14] Deribe B Woldemichael K Nemera G. Prevalence and factors influencing diabetic foot ulcer among diabetic patients attending arbaminch hospital, south Ethiopia. J Diabetes Metab (2014) 5(1):1–7. Available at: doi: 10.4172/2155-6156.1000322

[15] Amogne W Reja A Amare A. Diabetic foot disease in Ethiopian patients: a hospital-based study. Ethiopian J Health Dev (2011) 25(1):17–21. doi: 10.4314/ejhd.v25i1.69841

[16] Yimam A. Assessment of prevalence of diabetic foot ulcer and associated factors among diabetic patient attending tikur anbesa specialized hospital diabetic clinic, Addis Ababa, Ethiopia. (2017) 25 :17–21. Doctoral dissertation: http://etd.aau.edu.et/handle/123456789/6551?show=full

[17] Clinical and laboratory standard institutes (CLSI). Performance standards for antimicrobial susceptibility testing. 29th ed. CLSI supplement M100 Vol. 39. Wayne, PA: Clinical Laboratory Standards Institute (2020).

[18] Oates PJ. Polyol pathway and diabetic peripheral neuropathy. Int Rev Neurobiol (2002) 50:325–92. doi: 10.1016/s0074-7742(02)50082-9 12198816

[19] Kwon KT Armstrong DG. Microbiology and antimicrobial therapy for diabetic foot infections. Infect Chemother (2018) 50(1):11–20. doi: 10.3947/ic.2018.50.1.11 29637748 PMC5895826

[20] Murshed M. Bacteriological profile of diabetic foot infection and its effect on limb salvation. J Surg Sci (2020) 24(1):21–5. doi: 10.3329/jss.v24i1.52213

[21] Shah P Eswarawaka M Anne D Shah P Srivastava N. Bacteriological profile of diabetic foot. Int Surg J (2021) 8(2):704–9. doi: 10.18203/2349-2902.isj20210389

[22] Van Netten JJ Bus SA Apelqvist J Lipsky BA Hinchliffe RJ Game F. Definitions and criteria for diabetic foot disease. Diabetes/metabolism Res Rev (2020) 36:e3268. doi: 10.1002/dmrr.3268 31943705

[23] Ismail AA Meheissen MA Elaaty TAA Abd-Allatif NE Kassab HS. Microbial profile, antimicrobial resistance, and molecular characterization of diabetic foot infections in a university hospital. Germs (2021) 11(1):39–51. doi: 10.18683/germs.2021.1239 33898340 PMC8057848

[24] Dwedar R Ismail D Abdulbaky A. Diabetic foot infection: Microbiological causes with special reference to their antibiotic resistance pattern. Egyptian J Med Microbiol (2015) 24:95–102. doi: 10.12816/0024935

[25] Xie X Bao Y Ni L Liu D Niu S Lin H. Bacterial profile and antibiotic resistance in patients with diabetic foot ulcer in guangzhou, southern China: Focus on the differences among different wagner’s grades, IDSA/IWGDF grades, and ulcer types. Int J Endocrinol (2017), 8694903. doi: 10.1155/2017/8694903 29075293 PMC5623783

[26] Thanganadar Appapalam S Muniyan A Vasanthi Mohan K Panchamoorthy R. A study on isolation, characterization, and exploration of multiantibiotic-resistant bacteria in the wound site of diabetic foot ulcer patients. Int J Low Extrem Wounds (2021) 20(1):6–14. doi: 10.1177/1534734619884430 31735111

[27] Al Ayed MY Ababneh M Alwin Robert A Alzaid A Ahmed RA Salman A. Common pathogens and antibiotic sensitivity profiles of infected diabetic foot ulcers in Saudi Arabia. Int J Low Extrem Wounds (2018) 17(3):161–8. doi: 10.1177/1534734618793557 30141366

[28] Ponce de Leon A Merchant S Raman G Avendano E Chan J Tepichin Hernandez G. Pseudomonas infections among hospitalized adults in Latin America: a systematic review and meta-analysis. BMC Infect Dis (2020) 20(1):250. doi: 10.1186/s12879-020-04973-0 32220233 PMC7099820

[29] Mutonga DM Mureithi MW Ngugi NN Otieno FCF. Bacterial isolation and antibiotic susceptibility from diabetic foot ulcers in Kenya using microbiological tests and comparison with RT-PCR in the detection of s. aureus and MRSA. BMC Res Notes (2019) 12(1):244. doi: 10.1186/s13104-019-4278-0 31036061 PMC6489269

[30] Ogba OM Nisan E Eyam ES. Aerobic bacteria associated with diabetic foot ulcers and their susceptibility pattern. BioMed Dermatol (2019) 3:1. doi: 10.1186/s41702-019-0039-x

[31] Aleem S Multani H Bashir H. Bacteriological profile and antimicrobial sensitivity pattern of isolates from the diabetic foot of patients attending a teaching hospital in northern India. Asian J Med Sci (2021) 12(5):83–7. doi: 10.3126/ajms.v12i5.34415

[32] Akhi MT Ghotaslou R Asgharzadeh M Varshochi M Pirzadeh T Memar MY. Bacterial aetiology and antibiotic susceptibility pattern of diabetic foot infections in tabriz, Iran. GMS hygiene infection control (2015) 10:Doc02. doi: 10.3205/dgkh000245 25699225 PMC4332275

[33] Elbaz A Dhawan A Elramalli AK Algondi IA Anan A Elazomi A. Antimicrobial sensitivity patterns of pseudomonas aeruginosa isolates obtained from foot ulcer diabetes patients in Tripoli, Libya. 2018, Libyan Journal of Medical Research (LJMR), Vol : 12 (2)

[34] Miyan Z Fawwad A Sabir R Basit A. Microbiological pattern of diabetic foot infections at a tertiary care centre in a developing country. J Pak Med Assoc (2017) 67(5):665–9. ISSN: 0030-9982 28507348

[35] Adeyemo AT Kolawole B Rotimi VO Aboderin AO. Multicentre study of the burden of multidrug-resistant bacteria in the aetiology of infected diabetic foot ulcers. Afr J Lab Med (2021) 10(1):1261. doi: 10.4102/ajlm.v10i1.1261 33824857 PMC8008032

[36] Jain SK Barman R. Bacteriological profile of diabetic foot ulcer with special reference to drug-resistant strains in a tertiary care center in north-East India. Indian J Endocrinol Metab (2017) 21(5):688–94. doi: 10.4103/ijem.IJEM_546_16 PMC5628537 28989875

